# Kidney injury molecule-1: potential biomarker of acute kidney injury and disease severity in patients with COVID-19

**DOI:** 10.1007/s40620-021-01079-x

**Published:** 2021-06-10

**Authors:** Manuel J. Vogel, Julian Mustroph, Stephan T. Staudner, Simon B. Leininger, Ute Hubauer, Stefan Wallner, Christine Meindl, Frank Hanses, Markus Zimmermann, Lars S. Maier, Carsten G. Jungbauer, Julian Hupf

**Affiliations:** 1 Department of Internal Medicine II, University Hospital Regensburg, Franz-Josef-Strauss-Allee 11, 93053 Regensburg, Germany; 2 Department of Clinical Chemistry and Laboratory Medicine, University Hospital Regensburg, Franz-Josef-Strauss-Allee 11, 93053 Regensburg, Germany; 3 Emergency Department, University Hospital Regensburg, Franz-Josef-Strauss-Allee 11, 93053 Regensburg, Germany; 4 Department of Infection Prevention and Infectious Diseases, University Hospital Regensburg, Franz-Josef-Strauß-Allee 11, 93053 Regensburg, Germany

**Keywords:** KIM-1, COVID-19, NAG, Acute kidney injury

## Abstract

**Aims:**

The aim of the current study was to evaluate whether tubular markers kidney injury molecule-1 (KIM-1) and N-acetyl-ß-glucosaminidase (NAG) are related to acute kidney injury (AKI) and severe disease in patients with COVID-19.

**Methods and results:**

In this prospective observational clinical trial we examined a cohort of 80 patients with proof of acute respiratory infection and divided them into a COVID-19 cohort (n = 54) and a control cohort (n = 26). KIM-1 and NAG were measured from urine samples collected in the emergency department. We assessed the development of AKI, admission to the intensive care unit (ICU) and intrahospital death as clinical endpoints. Urinary KIM-1 and NAG were not significantly different between patients with SARS-CoV-2 and those with other respiratory infections (each p = n.s.). Eight patients from the COVID-19 cohort and five of the non-COVID-19-patients suffered from acute kidney injury during their stay. Nine COVID-19 patients and two non-COVID-19 patients were admitted to the ICU. KIM-1 was significantly elevated in COVID-19 patients with, compared to those without AKI (p = 0.005), as opposed to NAG and creatinine (each p = n.s.). Furthermore, KIM-1 was significantly elevated in the patients with COVID-19 that had to be transferred to the ICU (p = 0.015), in contrast to NAG and creatinine (each p = n.s.).

**Conclusion:**

Assessing KIM-1 in patients with COVID-19 might provide additional value in recognizing AKI at an early stage of disease. Further, KIM-1 might indicate higher risk for clinical deterioration as displayed by admission to the ICU.

**Graphical abstract:**

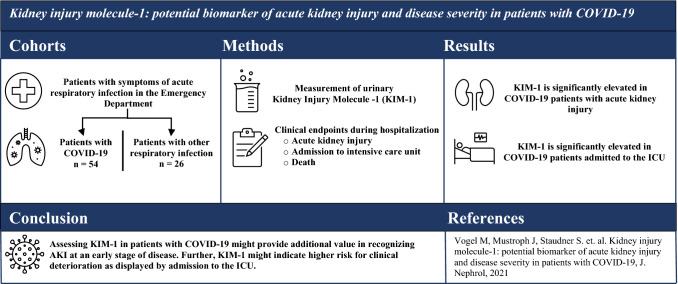

## Introduction

The COVID-19 pandemic is due to the rising number in cases of SARS-CoV-2 infection, and the tremendous number of deaths is a worldwide challenge regarding social as well as medical concerns. Limited health care resources and rapidly rising cases force us to find objective indicators for those at higher risk of severe disease. This will help to guarantee early and adequate medical treatment to those patients who need it most. Identifying those factors may help to achieve a deeper understanding of the pathophysiology of COVID-19 and to discover new ways of, for example, pharmacological treatment.

Kidney injury molecule-1 (KIM-1) and N-acetyl-ß-glucosaminidase (NAG) are both urinary markers of renal tubular damage [1, 2]. Previous studies have shown their benefit for early detection of acute kidney injury (AKI), outperforming serum creatinine for that purpose [3, 4]. Beyond that, urinary KIM-1 and NAG have been evaluated for their prognostic value regarding mortality and rehospitalization in patients with chronic heart failure [5]. Recently, KIM-1 was suggested as receptor for SARS-CoV-2 uptake in alveolar and proximal tubule epithelial cells [6].

While SARS-CoV-2 is especially known for its pulmonary manifestations, there is growing evidence of renal involvement in COVID-19. Several clinical trials have shown that acute kidney injury is highly prevalent and is associated with higher mortality rates in patients with COVID-19, especially in the critically ill [7–9]. However, it is still unknown whether KIM-1 and NAG are related to AKI and severe disease in patients with COVID-19 or other acute respiratory infections. Because of this, we analyzed a cohort of COVID-19 patients in this prospective observational clinical trial. To our knowledge, this is the first study to evaluate KIM-1 and NAG in the early stages of COVID-19.

## Methods

### Study population

Between March 2020 and February 2021, adult patients presenting with acute symptoms of respiratory infection (cough and/or fever) to the emergency department (ED) of the University Hospital Regensburg were included. Pre-specified exclusion criteria consisted of age below 18 years and the inability to understand and sign the declaration of consent. The ongoing study was approved by the Ethics Committee of the University of Regensburg. It was executed in alliance with the Good Clinical Practice guidelines and within the standards established for human experimentation by the Declaration of Helsinki.

**Fig. 1:**
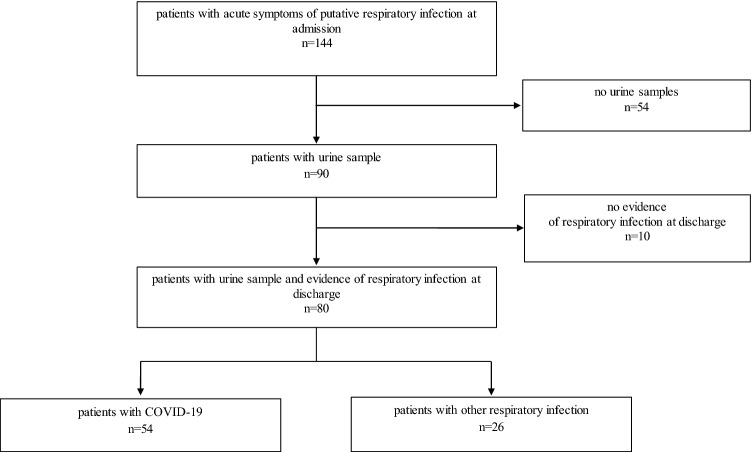
STARD flow diagram of the study design

Every patient was tested for SARS-CoV-2 infection by PCR analysis using throat rinse water or a nasopharyngeal swab. Patients with negative test results were used as the control group (defined as either bacterial or viral respiratory infection). The diagnosis at discharge was evaluated by a consultant physician to assure the correct distribution of patients in the control group. If there was no proof of respiratory infection, we excluded the case from analysis (n = 10, Fig. 1). A STARD flow diagram of the study design is displayed in Fig. 1 [10].

Baseline data, vital signs and findings of clinical examination were obtained and documented for all patients. To estimate the risk of acute clinical worsening we used the national early warning score 2 (NEWS-2). We assessed the development of AKI, admission to intensive care unit (ICU) and intrahospital death as clinical endpoints. The secondary endpoint was a composite of acute kidney injury, ICU-admission and death (defined as event). Acute kidney injury was diagnosed in accordance with the 2012 KDIGO criteria [11]. Estimated glomerular filtration rate (eGFR) was calculated by the CKD EPI formula from serum creatinine, sex and age [12].

### Sample procession and biochemical analysis

Blood and urine samples were collected in the emergency department directly after inclusion and sent to the central laboratory. Patients for whom urine samples were not available were excluded from this analysis (n = 54, Fig. 1). Together with the parameters of routine care, NT-proBNP, serum and urinary creatinine were measured immediately. Proteinuria was measured via turbidimetry with benzethonium chloride, and albuminuria by turbidimetry with anti-albumin-antibodies.

Urine samples were aliquoted in 500 µl cups after centrifugation and frozen at − 80 °C for later analysis of tubular markers KIM-1 and NAG. For the measurement, all samples were thawed at the same time, just before starting the assay, after which they were vortexed and centrifuged at 14,000 g. For analysis of KIM-1, the ELISA Duo kit and the respective ancillary reagent kit (R&D Systems, Minneapolis, Minnesota, USA) were used as intended in the manufacturer's instructions. The measurement of NAG was performed with a modified ELISA-assay in 96-well plates (Roche, Basel, Swiss). The wells were infused with 100μL of substrate solution and pre-incubated for 5 min at 37 °C. Afterwards, 5μL of either sample, control or standard was added to the plate, mixed carefully for 30 s and incubated for 60 min at 37 °C. The reaction was then stopped by adding 200μL of stop solution. Finally, after incubating for 10 min at 37 °C, absorbance was measured at 580 nm. To keep the dilutional bias of the urine samples as low as possible, all values of KIM-1 and NAG were related to urine creatinine (UCr). Distortion of the results due to UCr is unlikely as concentrations did not differ within the cohorts (each p > 0.05). Values of proteinuria and albuminuria were also normalized to urinary creatinine.

### Statistics

Variables were tested for normal distribution with the Kolmogorov-Smirnoff test. Student’s t-test was used for normally distributed continuous variables. Mann–Whitney-U test was utilized for not normally distributed, continuous values. Categorical variables were analyzed using Chi-square test or Fisher’s exact test. All p-values delineated are two tailed. Correlation coefficients were calculated according to Spearman. Normally distributed data is described as mean and standard deviation, whereas not normally distributed data is presented as median and interquartile range. To visualize differences in biomarker concentrations, boxplots were created. Furthermore, ROC analysis was performed and AUC and sensitivity, as well as specificity for KIM-1 and NAG were calculated. Statistical analysis was performed using SPSS 27 (SPSS Inc., Chicago, Illinois).

## Results

### Study population

We included 80 patients in the current study. Among them, 54 (67.5%) were tested positive for SARS-CoV-2, whereas 26 (32.5%) were classified as having either bacterial or viral respiratory infection. Clinical characteristics are listed in Table 1. In both groups, patients were predominantly male. The mean age was 56.8 years and did not significantly differ between the COVID-19 and the control cohort (60 vs. 55.3 years, p = n.s.). The controls suffered more often from chronic heart failure, coronary artery disease and chronic obstructive pulmonary disease than the SARS-CoV-2 cohort (each p < 0.05). Symptoms, vital signs and NEWS-2 did not differ between the two groups (each p = n.s.). Prescription of statins was higher among controls (p = 0.043), while the other baseline drugs did not differ between the cohorts (each p = n.s.).

**Table 1:** Baseline characteristics

	Overall collective	Controls	SARS-CoV-2	Statistics (p)
*Baseline characteristics*				
n	80	26	54	
Age^e^ (y)	56.8 ± 17.1	60 ± 16.8	55,3 ± 17.2	0.32^b^
Sex, % male (n)	66.3 (53)	73.1 (19)	63 (34)	0.37^a^
Smokers (continued), % (n)	11.3 (9)	19.2 (5)	7.4 (4)	0.12^a^
Intrahospital death, % (n)	5 (4)	3.8 (1)	5.6 (3)	0.74^a^
ICU admission, % (n)	13.8 (11)	7.7 (2)	16.7 (9)	0.27^a^
Acute Kidney Injury, % (n)	16.3 (13)	19.2 (5)	14.8 (8)	0.62^a^
Event (ICU/Death/AKI), % (n)	21.3 (17)	26.9 (7)	18.5 (10)	0.39^a^
CT scan, % (n)	87.5 (70)	88.5 (23)	87 (47)	0.86^a^
CT scan with CM, % (n)	31.3 (25)	38.5 (10)	27.8 (15)	0.35^a^
CM volume in CT scan^d^ (ml)	70 (70–77.5)	70 (70–90)	70 (70–70)	0.92^b^
*Baseline medication,* % (n)	70 (56)	69.2 (18)	70.4 (38)	0.74^a^
Immunosuppressants, % (n)	11.3 (9)	15.4 (4)	9.3 (5)	0.42^a^
Beta-blockers, % (n)	23.8 (19)	20.8 (8)	20.4 (11)	0.31^a^
ACE-/AT-1-inhibitors, % (n)	26.3 (21)	26.9 (7)	25.9 (14)	0.92^a^
Insulin, % (n)	5 (4)	3.8 (1)	5.6 (3)	0.74^a^
Metformin, % (n)	7.5 (6)	11.5 (3)	7.4 (4)	0.54^a^
Statins, % (n)	20 (16)	34.6 (9)	14.8 (8)	0.043^a^
Diuretics, % (n)	22.5 (18)	26.9 (7)	20.4 (11)	0.51^a^
ASS, % (n)	20.8 (16)	29.2 (7)	17 (9)	0.28^a^
*Pre-existing diseases*				
Coronary artery disease, % (n)	16.3 (13)	42.3 (11)	3.7 (2)	< 0.001^a^
Chronic heart failure, % (n)	6.3 (5)	15.4 (4)	1.9 (1)	0.019^a^
Arterial hypertension % (n)	45 (36)	57.7 (15)	38.9 (21)	0.11^a^
Diabetes mellitus, % (n)	16.3 (13)	23.1 (6)	13 (7)	0.25^a^
Obesity, % (n)	26.3 (21)	38.5 (10)	20.4 (11)	0.085^a^
COPD, % (n)	5 (4)	15.4 (4)	0 (0)	0.003^a^
Asthma, % (n)	2.5 (2)	0 (0)	3.7 (2)	0.32^a^
Chronic kidney disease, % (n)	13.8 (11)	15.4 (4)	13 (7)	0.77^a^
*Symptoms*				
Cough, % (n)	58.8 (47)	69.2 (18)	53.7 (29)	0.19^a^
Dyspnea, % (n)	57.5 (46)	65.4 (17)	53.7 (29)	0.32^a^
Fever, % (n)	65 (52)	69.2 (18)	63 (34)	0.58^a^
Chills, % (n)	50 (40)	50 (13)	50 (27)	> 0.99^a^
Fatigue, % (n)	76.3 (61)	65.4 (17)	81.5 (44)	0.11^a^
Anosmia, % (n)	12.5 (10)	3.8 (1)	16.7 (9)	0.10^a^
Dysgeusia, % (n)	31.1 (25)	19.2 (5)	37 (20)	0.11^a^
*Vital signs*				
Heart rate^e^ (b.p.m.)	90 ± 18.5	96 ± 25	87 ± 14.5	0.096^c^
Systolic blood pressure^e^ (mmHg)	130 ± 18	129 ± 21	130.5 ± 17	0.90^c^
Diastolic blood pressure^e^ (mmHg)	79.5 ± 12.5	79 ± 14	79 ± 12	0.92^c^
Oxygen demand, % (n)	41.3 (33)	42.3 (11)	40.7 (22)	0.89^a^
Temperature^e^(°C)	37.5 ± 0.84	37.54 ± 0.98	37.49 ± 0.78	0.64^c^
Respiratory rate^e^ (/min)	22.5 ± 7	22.5 ± 7	22 ± 7	0.49^c^
NEWS2^d^	4 (1.25–6)	4 (1–6)	3.5 (2–6)	0.72^b^
*Biomarkers*				
KIM-1^d^ (ng/g UCr)	1316 (490–2480)	1468 (420–2923)	1316 (485–2316)	0.55^b^
NAG^d^ (U/g UCr)	4.75 (1.54–10.7)	5.1 (1.5–13.3)	4.8 (1.5–10.5)	0.75^b^
NT-proBNP^d^ (pg/ml)	142 (50–670.25)	393.5 (50–1553.5)	103 (50–508)	0.085^b^
Serum creatinine^d^ (mg/dl)	0.95 (0.77–1.26)	0.97 (0.79–1.17)	0.95 (0.76–1.29)	0.68^b^
eGFR^d^ (ml/min per m^2^)	84 (55–98.5)	81.5 (61.25–97)	86.5 (53.5–100)	0.58^b^
Proteinuria^d^ (mg/g UCr)	154 (90–442,5)	148 (87–487)	154 (90.5–416.5)	0.98^b^
IL-6^d^ (pg/ml)	36 (13.4–67.6)	59.2 (26.8–260)	24.6 (13.1–60.5)	0.012^b^
CRP^d^ (mg/dl)	43.1 (15–77.5)	52.2 (8–85.3)	38.8 (20.55–77)	0.95^b^
White blood cell count^d^ (n/nl)	6.89 (4.69–10.94)	10.6 (7.32–14.88)	5.23 (4.15–8.6)	< 0.001^b^

*ACE* angiotensin–converting enzyme; *AT-1* angiotensin II receptor type I; *CM* contrast media; *COPD* chronic obstructive pulmonary disease; *CRP* C-reactive protein; *CT scan* computed tomography scan; *eGFR* estimated glomerular filtration rate; *ICU* intensive care unit; *IL-6* interleukin 6; *KIM-1* kidney injury molecule 1; *NAG* N-acetyl-β-glucoasaminidase; *NEWS-2* national early warning score 2; *NT-proBNP* N-terminal prohormone of brain natriuretic peptide

^a^Fisher´s exact test

^b^Mann-Whitney-U–test

^c^Student’s t-test

^d^Median (interquartile range)

^e^Mean ± standard deviation;

Nine (16.7%) patients with COVID-19 were admitted to the ICU, three (5.6%) of them died because of the infection. Regarding the control group, one (3.8%) patient died, and two (7.7%) were admitted to the ICU. Eight (14.8%) patients from the SARS-CoV-2 group and five (19.2%) of the non-COVID-19 patients suffered from acute kidney injury during their stay. In total, ten (18.5%) COVID-19-patients and seven (26.9%) non-COVID-19 patients suffered from at least one of the events considered in the composite endpoint. The median interval time between presentation to the emergency department and the occasion of acute kidney injury was 4 days in the COVID-19 cohort and 3 days in the control cohort. The two patients in the control group and the five patients in the COVID-19 cohort were transferred to the ICU on the day of hospital admission. The other three COVID-19 patients were admitted to the ICU between the third and the fifth day after hospitalization. The median time between hospital admission and death was 13 days in the COVID-19 cohort. The control patient died only a few hours after admission. For further information see also Table 2.

**Table 2:** Median interval time (days) between presentation to the ED and occurrence of AKI/admission to ICU/death

	Controls	SARS-CoV-2	Statistics (p)
AKI^b^	3 (1–3)	4 (1–5)	0.52^a^
ICU^b^	0 (0–0)	0 (0–3)	0.53^a^
Death^b^	0 (0–0)	13 (8–13)	0.50^a^

AKI = acute kidney injury, *ICU* intensive care unit, *ED* emergency department

^a^Mann-Whitney-U–test

^b^Median (interquartile range)

KIM-1 and NAG, as well as serum creatinine, eGFR, proteinuria and albuminuria were not significantly different between patients with SARS-CoV-2 and those with other respiratory infections (each p = n.s.). Interleukin-6 (IL-6) as well as white blood cell count were significantly higher in the control cohort than in the COVID-19 cohort (each p < 0.05). There were no differences between the two cohorts regarding CRP and NT-proBNP (each p = n.s.).

KIM-1 was significantly correlated with NAG in the COVID-19 cohort (ρ = 0.63, p < 0.001) as well as in the control cohort (ρ = 0.66, p < 0.001). Both KIM-1 and NAG were significantly negatively correlated with eGFR in the COVID-19 cohort (KIM-1: ρ = -0.43,s NAG: ρ = -0.34, each p < 0.05), as opposed to serum creatinine (each p = n.s.). In the control cohort, only NAG was significantly negatively correlated with eGFR (ρ = -0.45, p < 0.05), in contrast to KIM-1 (p = n.s.). Neither KIM-1 nor NAG correlated with serum creatinine in the control group (each p = n.s.). KIM-1 and NAG were significantly correlated with level of proteinuria in the COVID-19 cohort (KIM-1: ρ = 0.54, NAG: ρ = 0.68, each p < 0.001) and in the control cohort (KIM-1: ρ = 0.61, p < 0.001, NAG: ρ = 0.59, p < 0.05).

### Acute kidney injury

**Fig. 2:**
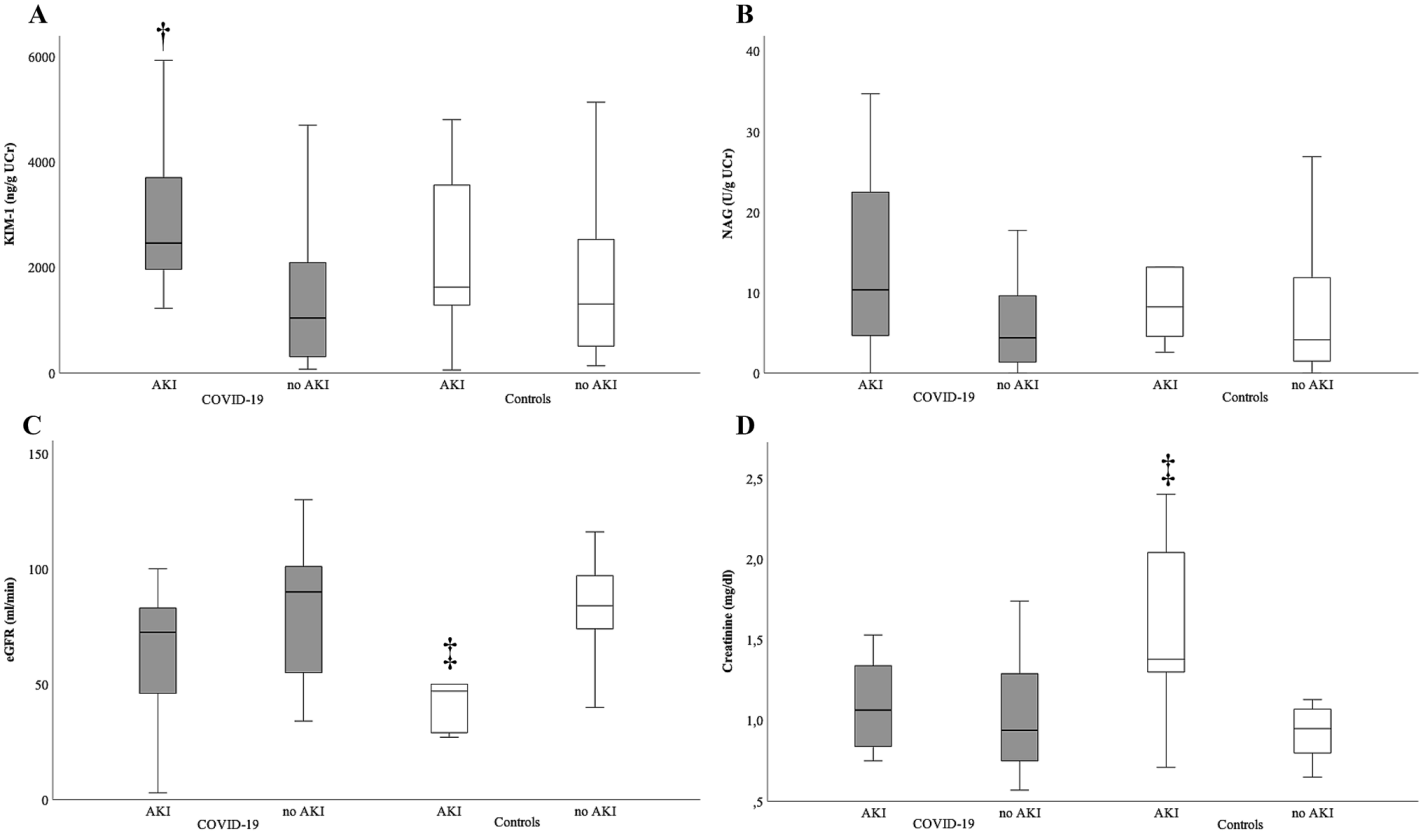
Boxplots showing KIM-1, NAG, serum creatinine and eGFR of patients with and without AKI in the COVID-19 study cohort (displayed in gray color) and the control cohort (white color). **A** COVID-19: KIM-1 is significantly elevated in patients who suffer acute kidney injury (†: p < 0.05 vs. no AKI (COVID-19)). Controls: Patients with AKI show higher values of KIM-1 without reaching statistical significance. **B** COVID-19/Controls: NAG shows a trend towards higher concentrations in patients with AKI. **C** COVID-19: eGFR shows a trend towards lower levels. Controls: eGFR is significantly decreased in patients with AKI versus no AKI (‡: p < 0.05 vs. no AKI (Controls)). **D** COVID-19: Creatinine shows a trend towards higher values in patients with AKI versus without AKI. Controls: Creatinine is significantly elevated in patients reaching the composite endpoint (‡: p < 0.05 vs. no AKI (Controls))

Regarding patients with COVID-19, KIM-1 levels were significantly higher in patients with, compared to those without AKI (p = 0.005, Fig. 2A). NAG showed a not statistically significant trend to be elevated in COVID-19 patients suffering from acute kidney injury (p = 0.086; Fig. 2B).

According to ROC analysis, KIM-1 was able to detect acute kidney injury in COVID-19 subjects with an AUC of 0.81 (p = 0.006, Fig. 4A). Further information regarding ROC analysis can be found in Table 3.

**Table 3:** Median concentration and ROC analysis of KIM-1 and NAG for detection of AKI, ICU admission and the composite endpoint in the COVID-19 cohort

	KIM-1	NAG
AKI vs. no AKI		
Concentration^a^	2460 vs. 1040 [ng/g UCr]	10.3 vs. 4.4 [U/g UCr]
ROC analysis		
Cut-off value / sensitivity / specificity	1590 [ng/g UCr] / 87.5% / 65%	7.2 [U/g UCr] / 62.5% / 67.5%
ICU vs. no ICU		
Concentration^a^	2195 vs. 1040 [ng/g UCr]	10.7 vs. 3.65 [U/g UCr]
ROC analysis		
Cut-off value / sensitivity / specificity	1590 [ng/g UCr] / 78% / 64%	7.2 [U/g UCr] / 67% / 69%
ICU/Death/AKI vs no ICU/Death/AKI		
Concentration^a^	2235 vs. 934 [ng/g UCr]	9 vs. 4.4 [U/g UCr]
ROC analysis		
Cut-off value / sensitivity / specificity	1590 [ng/g UCr] / 80% / 66%	7.2 [U/g UCr] / 60% / 68%

*AKI* acute kidney injury; *ICU* intensive care unit; *KIM-1* kidney injury molecule-1; *NAG* N-acetyl-β-glucoasaminidase; *UCr* urinary creatinine

^a^Median

In the COVID-19 cohort there was a not significant trend towards higher concentrations of serum creatinine and lower values of eGFR in patients with, compared to those without AKI (each p = n.s.). Proteinuria and albuminuria were significantly higher in COVID-19 patients who developed AKI compared to those who did not (each p < 0.05, Table 4). Regarding the detection of acute kidney injury, ROC analysis showed an AUC of 0.78 for proteinuria and an AUC of 0.73 for albuminuria (each p < 0.05). Additional information concerning the ROC analysis is presented in Table 5.

**Table 4:** Proteinuria characteristics

	Proteinuria [mg/gUCr]	Statistics (p)	Albuminuria [mg/gUCr]	Statistics
AKI vs. no AKI				
Controls^b^	373 vs. 131	0.41^a^	193 vs. 37	0.18^a^
SARS-CoV-2^b^	461 vs. 142	0.011^a^	93 vs. 26	0.04^a^
ICU vs. no ICU				
Controls^b^	1089 vs. 126	0.26 ^a^	768 vs. 41	0.81^a^
SARS-CoV-2^b^	460 vs. 146	0.059 ^a^	90 vs. 26	0.09^a^

*AKI* acute kidney injury; *ICU* intensive care unit; *UCr* Urinary creatinine

^a^Mann-Whitney-U–test

^b^Median

**Table 5:** ROC analysis of proteinuria and albuminuria for the detection of AKI in COVID-19 patients

	Proteinuria	Albuminuria
Cut-off value	200 [mg/g UCr]	42 [mg/g UCr]
Sensitivity	75%	75%
Specificity	65%	61%

*AKI* acute kidney injury; *ROC* receiver operating curve

Regarding patients without SARS-CoV-2-infection, KIM-1 as well as NAG showed a non-significant trend to be higher in patients with AKI (each p = n.s.; Fig. 2A, B). Serum creatinine was significantly higher and eGFR significantly lower in AKI patients in the control cohort (each p < 0.05, Fig. 2C, D).

### Admission to the intensive care unit

**Fig. 3:**
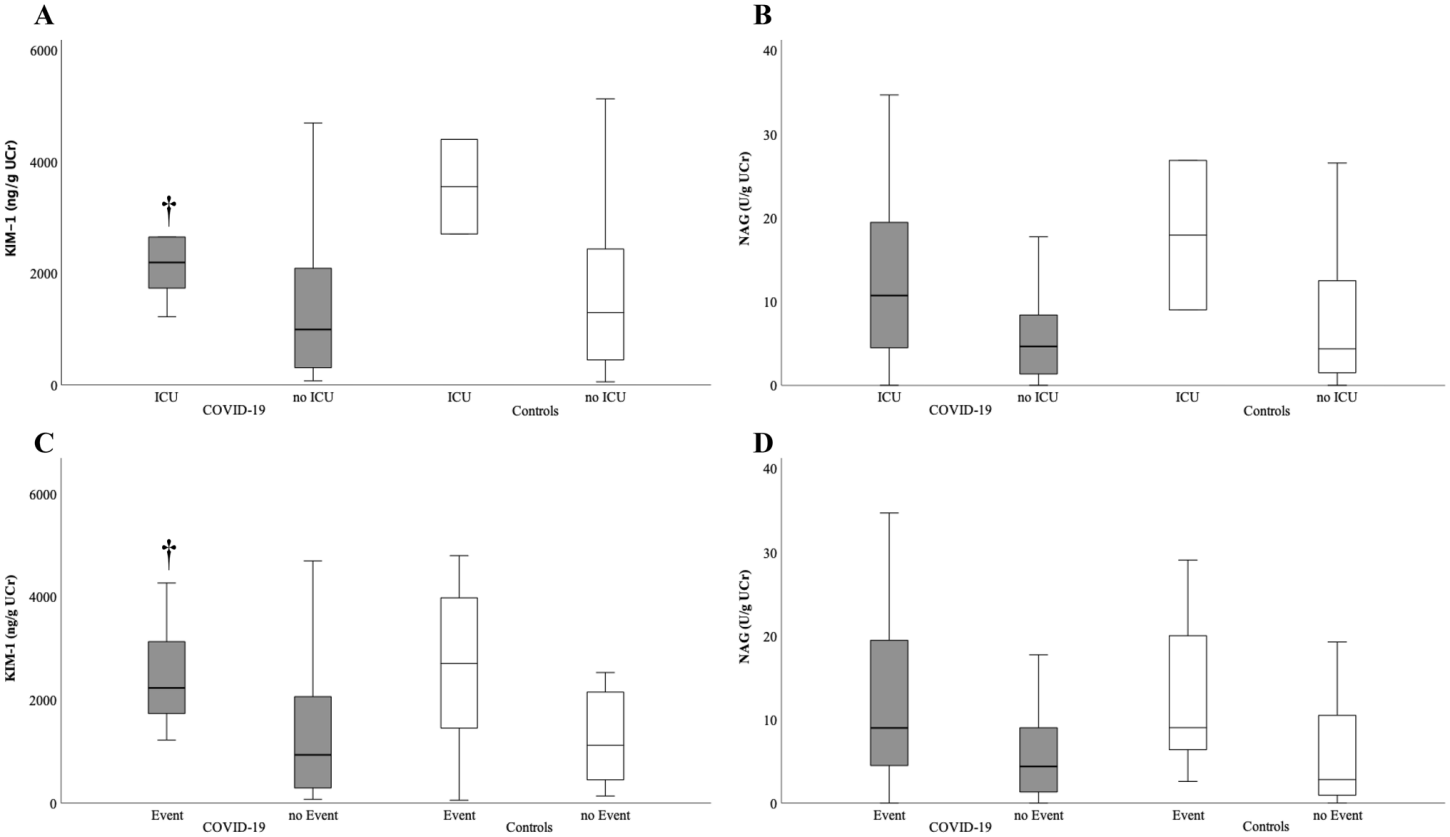
Boxplots showing KIM-1 and NAG in patients who were admitted to ICU (above) or reached the composite endpoint (below) in COVID-19 study cohort (displayed in gray color) and the control cohort (white color). **A** COVID-19: KIM-1 is significantly elevated in patients who had to be treated in ICU (†: p < 0.05 vs. no ICU (COVID-19)). Controls: Patients admitted to the ICU show higher values of KIM-1 without reaching statistical significance. **B** COVID-19/Controls: NAG shows a trend towards higher concentrations in patients admitted to ICU. **C** COVID-19: KIM-1 is significantly elevated in patients suffering an event (ICU-admission, Death and/or AKI) subsequently to their infection (†: p < 0.05 vs. no event (COVID-19)). Controls: KIM-1 shows a statistically not significant trend towards higher concentrations in patients suffering an event compared to those who don’t. **D** COVID-19/Controls: NAG shows a trend towards higher values in patients suffering an event

**Fig. 4:**
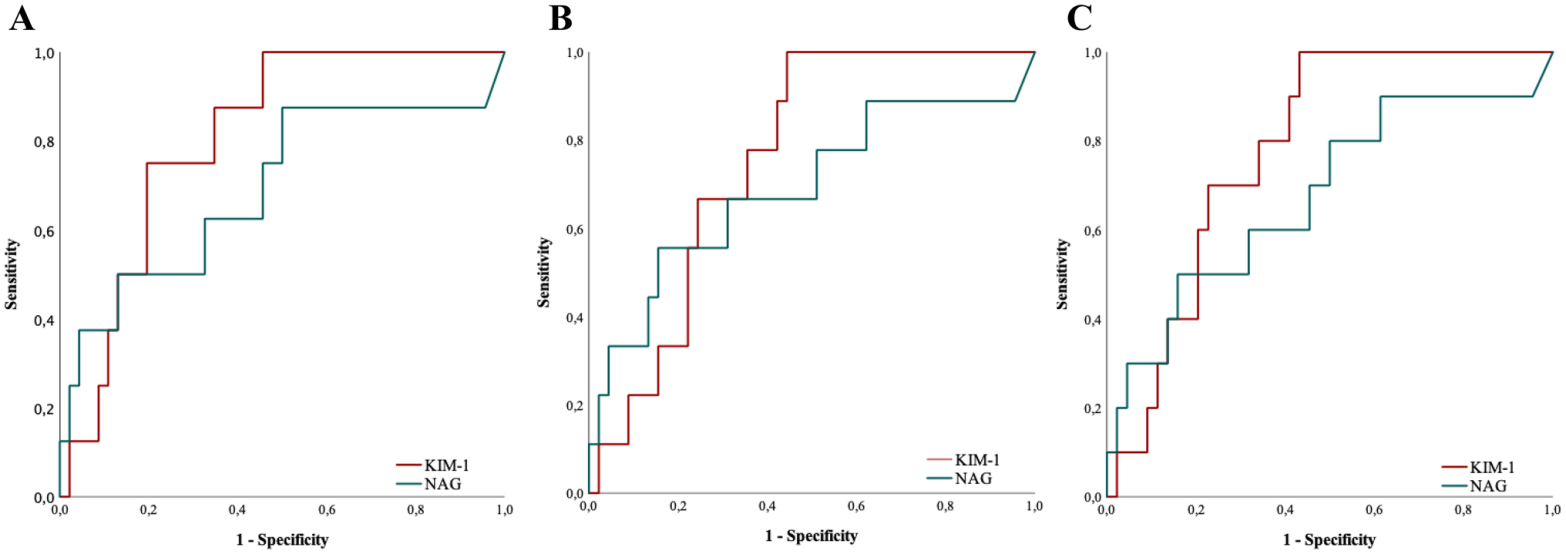
Receiver operating characteristic analysis of urinary KIM-1 and NAG in COVID-19 cohort. **A** Predictive values of KIM-1 and NAG for AKI vs. no AKI in COVID-19 cohort. Area under the curve KIM-1: 0.81 (cut-point of 1590 ng/g UCr: sensitivity: 87.5%, specificity: 65%, p < 0.001) as well as NAG: 0.69 (p = n.s.). **B** Predictive values of KIM-1 and NAG for ICU vs. no ICU in COVID-19 cohort. Area under the curve KIM-1: 0.76 (cut-point of 1590 ng/g UCr: sensitivity: 79%, specificity: 64%, p = 0.015) as well as NAG: 0.69 (p = n.s.). **C** Predictive values of KIM-1 and NAG for composite endpoint (AKI/ICU-admission/death) in COVID-19 cohort. Area under the curve KIM-1: 0.78 (cut-point of 1590 ng/g UCr: sensitivity: 80%, specificity: 66%, p = 0.006) as well as NAG: 0.68 (p = n.s.)

In patients with COVID-19, KIM-1 was significantly higher in patients admitted to the ICU (p = 0.015, Fig. 3A). NAG showed a non-significant trend towards higher concentrations in ICU versus non-ICU patients (p = 0.072, Fig. 3B). Regarding the prediction of subsequent ICU admission, ROC analysis showed an AUC of 0.76 for KIM-1 (Fig. 4B).

KIM-1 was significantly correlated with NEWS-2 in the COVID-19 cohort (ρ = 0.457, p = 0.001) as well as in the control cohort (ρ = 0.607, p = 0.002), as opposed to NAG (each p = n.s.)

In the control cohort, both KIM-1 and NAG showed a statistically non-significant trend to be elevated in patients who had to be transferred to the ICU (each p = n.s., Fig. 3A and B).

In the COVID-19 cohort as well as in the control cohort, there was a non-significant trend towards higher concentrations of serum creatinine and lower values of eGFR in ICU versus non-ICU patients (each p = n.s.).

Proteinuria and Albuminuria showed a not significant trend towards higher values in patients that had to be treated in the ICU in both cohorts (p = n.s., Table 4).

### Composite endpoint ICU/Death/AKI

Regarding patients with COVID-19, KIM-1 was observed in significantly higher concentrations in patients reaching the combined endpoint (= 0.006, Fig. 3C). NAG showed a non-significant trend towards higher concentrations in patients reaching the composite endpoint (p = 0.08, Fig. 3D). Regarding the detection of the composite endpoint, ROC analysis showed an AUC of 0.78 for KIM-1 (p = 0.006; Fig. 4C).

KIM-1 and NAG showed a statistically non-significant trend to be elevated in patients reaching the composite endpoint in the control group (p = n.s., Fig. 2C).

In the COVID-19 cohort as well as in the control cohort, there was a non-significant trend towards higher concentrations of serum creatinine and lower values of eGFR in patients reaching the combined endpoint (each p = n.s.).

## Discussion

In the current study we evaluated the relationship of tubular markers KIM-1 and NAG to the severity of disease and the occurrence of acute kidney injury in COVID-19 and other respiratory infections.

For the first time it has been shown that KIM-1 is significantly elevated on the day of hospital admission in patients with COVID-19 suffering from acute kidney injury during hospitalization. Furthermore, KIM-1 detected following AKI in COVID-19 patients with a good predictive value and was significantly associated with future admission to the intensive care unit with a satisfying predictive value. Moreover, we found significantly higher values of KIM-1 in patients who reached the composite endpoint, consisting of acute kidney injury, ICU admission and death. These findings might provide additional value regarding risk evaluation in patients with COVID-19.

Proteinuria and albuminuria were also significantly elevated in COVID-19 patients with later occurrence of AKI, which concurs with the findings of previous studies [13, 14]. However, compared to proteinuria and albuminuria, KIM-1 detected subsequent AKI with higher sensitivity and specificity. Further, KIM-1 showed a trend towards higher AUC values in ROC analysis. These findings implicate that KIM-1 may be superior to proteinuria and albuminuria regarding early detection of AKI. One explanation for these findings may be that the level of proteinuria is also highly influenced by chronic kidney disease and other pre-existing conditions [15]. Furthermore, proteinuria is highly prevalent in patients with COVID-19, even in those without AKI [16]. However, proteinuria and albuminuria did not differ between patients with and without COVID-19 in this study.

Furthermore, only KIM-1, but not proteinuria and albuminuria, was significantly elevated in COVID-19 patients who needed treatment in the ICU compared to those who did not. Therefore, KIM-1 may be superior to proteinuria and albuminuria regarding risk evaluation in COVID-19.

Due to the small number of patients, we can only put forth these hypotheses. Further studies with larger cohorts are needed to evaluate the extent to which KIM-1 outmatches proteinuria and albuminuria in the detection of AKI and later ICU admission in COVID-19.

### Detection of acute kidney injury in COVID-19

Acute kidney injury is highly prevalent in patients with COVID-19 and is associated with high mortality rates [7–9]. Therefore, the early diagnosis and treatment of acute kidney injury in COVID-19 may be essential to reduce morbidity and mortality rates [17, 18]. Renal function is usually assessed by serum creatinine, which predominantly displays glomerular function [5]. However, it is a poor indicator for tubular function and is subsequently unable to detect acute damage, which is mainly located in tubular epithelium [4, 5]. Therefore, serum creatinine is considered to be a relatively late indicator of AKI, which is neither sensitive nor specific for the detection of acute kidney injury [17, 19, 20].

In contrast, tubular markers KIM-1 and NAG are considered to be early and sensitive indicators of acute kidney injury [19, 20]. KIM-1 is a type 1 transmembrane glycoprotein, mainly expressed in renal tubular epithelium [3, 4]. In acute kidney injury, KIM-1 expression is highly upregulated, leading to abundant amounts of KIM-1 in the urine [3, 4]. NAG on the other hand is a lysosomal enzyme, which is highly prevalent in renal proximal tubule cells [4]. Urinary NAG reflects tubular lesions, as glomerular filtration is precluded due to its large molecule mass [4]. In healthy individuals, only very small amounts of the tubular markers can be found in the urine [3, 4]. KIM-1 is usually detectable within 24 h after acute kidney injury [21]. The exact onset of NAG increase after AKI is still unclear, but it was found to be elevated in children within 6 h after coronary angiography [22]. Thus, both markers are able to detect AKI early, especially at times where serum creatinine and GFR are still unchanged [4]. Tu et al. examined a cohort of severe septic patients admitted to the ICU and found that in patients who developed AKI, KIM-1 levels were elevated within 6 h of ICU admission [23]. Interestingly, the first rise in serum creatinine concentration was detected 24 h later [23]. Regarding prognostic value, KIM-1 and NAG were able to predict the severity as well as adverse outcomes, such as dialysis and death, in patients with AKI [19, 24].

Similar to these findings, the results of the current study imply that KIM-1 may be able to predict AKI in patients with COVID-19 and that KIM-1 may be superior to NAG for that purpose. However, further research in a larger cohort is needed to confirm our findings.

### Mechanisms of acute kidney injury in COVID-19

The exact pathophysiology of acute kidney injury in COVID-19 is still not fully understood and presumably an interaction of SARS-CoV-2- specific and rather unspecific mechanisms [7].

First of all, SARS-CoV-2 uses the ACE2 receptor for cell entry, which is highly expressed in the kidneys, especially in the apical brush borders of proximal tubules as well as in podocytes [25–27]. Beyond that, KIM-1 was suggested to be a receptor for SARS-CoV-2, which is also highly expressed in proximal tubule cells [6]. Virus incorporation may lead to podocyte dysfunction as well as acute proximal tubular damage and necrosis, which is concurrent with our findings of significantly elevated KIM-1 levels in COVID-19 patients with AKI [7]. In contrast, Rossi et al. found neither SARS-CoV-2 nor typical viral cytopathic effects in the kidney biopsy of a COVID-19 patient with acute kidney injury [28]. Thus, the role of viral tropism in acute kidney injury remains unclear and needs further evaluation.

Besides a putative cytopathic effect, the SARS-CoV-2-induced down regulation of membrane-bound ACE2, which degrades angiotensin II to angiotensin (1–7), may lead to the accumulation of angiotensin II and subsequently to inflammation, vasoconstriction and fibrosis in the kidney [29]. This hypothesis is also strengthened by Su et al., who found specific virus-like particles as well as prominent ACE2 expression particularly in acutely injured proximal tubule cells [30].

Moreover, SARS-COV-2 leads to an excessive release of inflammatory cytokines, especially in critically ill patients [31, 32]. Particularly IL-6 was found to play a role in the pathophysiology of acute kidney injury in earlier studies and may contribute to AKI in COVID-19 as well [33]. Interestingly, in the current study IL-6 was significantly higher in COVID-19 patients with AKI compared to IL-6 levels in those without AKI (data not shown).

There are also a few nonspecific factors that may contribute to AKI in patients with COVID-19. First of all, the prescription of nephrotoxic medications, as well as the use of radiographic contrast media to detect pulmonary embolism, which is highly prevalent in COVID-19 [7, 34]. In the current study, contrast media exposure did not differ between the COVID-19 cohort and the control cohort. Further, besides two patients in the control cohort and two in the COVID-19 cohort who received furosemide prior to AKI, no nephrotoxic drugs were prescribed.

Beyond that, kidney congestion due to right heart failure and renal hypoperfusion due to low cardiac output and hypovolemia may also contribute to AKI [17, 18]. However, acute heart failure did not relevantly contribute to AKI in the current study.

### Relevance of tubular markers in COVID-19

Assessing KIM-1 in patients with COVID-19 might provide additional value in recognizing acute kidney injury at an early stage of disease. Furthermore, KIM-1 may be helpful in identifying high risk patients for serious renal involvement in COVID-19. Those patients may benefit from an adaptation in their treatment, for example by avoiding nephrotoxins and intensifying surveillance of urine output, volume status and other hemodynamic parameters. Consequently, the probability of a favorable outcome may be enhanced.

However, due to the small number of events in the current study cohorts, no reliable Cox or logistic regression model for further analysis of the prognostic values of KIM-1 could be performed [35, 36]. Therefore, we can only generate these hypotheses. Beyond that, high levels of KIM-1 indicate a high risk for future admission to the intensive care unit. With limited health care resources and huge incidences of COVID-19, it is crucial to identify those high-risk patients and to guarantee adequate treatment for patients who need it the most.

Further research with larger cohorts is urgently needed to confirm our findings and evaluate the relevance of the tubular markers in COVID-19.

### Limitations

Only a small number of patients could be included in our study. Especially in the control cohort, the lack of a statistically significant difference between KIM-1 levels in patients who did or did not reach one of the endpoints may be due to the small sample size. For the same reason no reliable Cox or logistic regression model could be performed. Therefore, a larger cohort will be needed to affirm the current data. Furthermore, urine sample collection was limited due to infection-caused hypovolemia. Also, no renal biopsies were performed. Therefore, there is no histopathological confirmation of acute tubular injury as displayed by elevated KIM-1 levels.

## References

[1] Price RG (1992). The role of NAG (N-acetyl-beta-D-glucosaminidase) in the diagnosis of kidney disease including the monitoring of nephrotoxicity. Clin Nephrol.

[2] Ichimura T, Bonventre JV, Ronique Bailly V, Wei H, Hession CA, Cate RL (1998). Kidney Injury Molecule-1 (KIM-1), a putative epithelial cell adhesion molecule containing a novel immunoglobulin domain, is up-regulated in renal cells after injury. J Biol Chem.

[3] Moresco RN, Bochi GV, Stein CS, De Carvalho JAM, Cembranel BM, Bollick YS (2018). Urinary kidney injury molecule-1 in renal disease. Clin Chim Acta.

[4] Andreucci M, Faga T, Pisani A, Perticone M, Michael A (2017). The ischemic/nephrotoxic acute kidney injury and the use of renal biomarkers in clinical practice. Eur J Intern Med.

[5] Jungbauer CG, Birner C, Jung B, Buchner S, Lubnow M, Von Bary C (2011). Kidney injury molecule-1 and N-acetyl-ß-d-glucosaminidase in chronic heart failure: possible biomarkers of cardiorenal syndrome. Eur J Heart Fail.

[6] Yang C, Zhang Y, Zeng X, Chen H, Chen Y, Yang D (2021). Kidney injury molecule-1 is a potential receptor for SARS-CoV-2. J Mol Cell Biol.

[7] Gabarre P, Dumas G, Dupont T, Darmon M, Azoulay E, Zafrani L (2020). Acute kidney injury in critically ill patients with COVID-19. Intensive Care Med.

[8] Hirsch JS, Ng JH, Ross DW, Sharma P, Shah HH, Barnett RL (2020). Acute kidney injury in patients hospitalized with;COVID-19. Kidney Int.

[9] Hansrivijit P, Qian C, Boonpheng B, Thongprayoon C, Vallabhajosyula S, Cheungpasitporn W (2020). Incidence of acute kidney injury and its association with mortality in patients with COVID-19: a meta-analysis. J Investig Med.

[10] Bossuyt PM, Reitsma JB, Bruns DE, Gatsonis CA, Glasziou PP, Irwig L (2015). STARD 2015: an updated list of essential items for reporting diagnostic accuracy studies. BMJ.

[11] Kidney Disease: Improving Global Outcomes (KDIGO) Acute Kidney Injury Work Group (2012). KDIGO clinical practice guideline for acute kidney injury. Kidney Int.

[12] Levey AS, Stevens LA, Schmid CH, Zhang Y, Castro AF, Feldman HI (2009). A new equation to estimate glomerular filtration rate. Ann Intern Med.

[13] Yildirim C, Ozger HS, Yasar E, Tombul N, Gulbahar O, Yildiz M (2021). Early predictors of acute kidney injury in COVID-19 patients. Nephrology.

[14] Karras A, Livrozet M, Lazareth H, Benichou N, Hulot J-S, Fayol A (2021). Proteinuria and clinical outcomes in hospitalized COVID-19 Patients. Clin J Am Soc Nephrol.

[15] Haynes J, Haynes R (2006). Proteinuria. BMJ.

[16] Mohamed MMB, Velez JCQ (2021). Proteinuria in COVID-19. Clin Kidney J.

[17] Ronco C, Bellomo R, Kellum JA (2019). Acute kidney injury. Lancet.

[18] Ronco C, Reis T, Husain-Syed F (2020). Management of acute kidney injury in patients with COVID-19. Lancet Respir Med.

[19] Coca SG, Yalavarthy R, Concato J, Parikh CR (2008). Biomarkers for the diagnosis and risk stratification of acute kidney injury: a systematic review. Kidney Int.

[20] Schrier RW, Wang W, Poole B, Mitra A (2004). Acute renal failure: Definitions, diagnosis, pathogenesis, and therapy. J Clin Investig.

[21] Huang Y, Craig D-W (2011). The clinical utility of kidney injury molecule 1 in the prediction, diagnosis and prognosis of acute kidney injury: a systematic review. Inflamm Allergy Drug Targets.

[22] Benzer M, Alpay H, Baykan Ö, Erdem A, Demir IH (2016). Serum NGAL, cystatin C and urinary NAG measurements for early diagnosis of contrast-induced nephropathy in children. Ren Fail.

[23] Tu Y, Wang H, Sun R, Ni Y, Ma L, Xv F (2014). Urinary netrin-1 and KIM-1 as early biomarkers for septic acute kidney injury. Ren Fail.

[24] Liangos O, Perianayagam MC, Vaidya VS, Han WK, Wald R, Tighiouart H (2007). Urinary N-acetyl-β-(D)-glucosaminidase activity and kidney injury molecule-1 level are associated with adverse outcomes in acute renal failure. J Am Soc Nephrol.

[25] Hamming I, Timens W, Bulthuis M, Lely AT, Navis GJ, Van Goor H (2004). Rapid communication tissue distribution of ACE2 protein, the functional receptor for SARS coronavirus. A first step in understanding SARS pathogenesis. J Pathol.

[26] Zou X, Chen K, Zou J, Han P, Hao J, Han Z (2020). Single-cell RNA-seq data analysis on the receptor ACE2 expression reveals the potential risk of different human organs vulnerable to 2019-nCoV infection. Front Med.

[27] Hoffmann M, Kleine-Weber H, Schroeder S, Krüger N, Herrler T, Erichsen S (2020). SARS-CoV-2 cell entry depends on ACE2 and TMPRSS2 and is blocked by a clinically proven protease inhibitor. Cell.

[28] Rossi GM, Delsante M, Pilato FP, Gnetti L, Gabrielli L, Rossini G (2020). Kidney biopsy findings in a critically ill COVID-19 patient with dialysis-dependent acute kidney injury: a case against “SARS-CoV-2 Nephropathy”. Kidney Int Rep.

[29] Vaduganathan M, Vardeny O, Michel T, Mcmurray JJV, Pfeffer MA, Solomon SD (2020). Renin-angiotensin-aldosterone system inhibitors in patients with Covid-19. N Engl J Med.

[30] Su H, Yang M, Wan C, Yi LX, Tang F, Zhu HY (2020). Renal histopathological analysis of 26 postmortem findings of patients with COVID-19 in China. Kidney Int.

[31] Huang C, Wang Y, Li X, Ren L, Zhao J, Hu Y (2020). Clinical features of patients infected with 2019 novel coronavirus in Wuhan. China Lancet.

[32] Zhou F, Yu T, Du R, Fan G, Liu Y, Liu Z (2020). Clinical course and risk factors for mortality of adult inpatients with COVID-19 in Wuhan, China: a retrospective cohort study. Lancet.

[33] Su H, Lei CT, Zhang C (2017). Interleukin-6 signaling pathway and its role in kidney disease: an update. Front Immunol.

[34] Helms J, Tacquard C, Severac F, Leonard-Lorant I, Ohana M, Delabranche X (2020). High risk of thrombosis in patients with severe SARS-CoV-2 infection: a multicenter prospective cohort study. Intensive Care Med.

[35] Peduzzi P, Concato J, Kemper E, Holford TR, Feinstein AR (1996). a simulation study of the number of events per variable in logistic regression analysis. J Clin Epidemiol.

[36] Austin PC, Allignol A, Fine JP (2017). The number of primary events per variable affects estimation of the subdistribution hazard competing risks model. J Clin Epidemiol.

